# On Mucous or Catarrhal Pneumonia in Children

**Published:** 1850-08

**Authors:** 


*On Mucous or Catarrhal Pneumonia in Children.* By M. Duclos.—M. Duclos observes, that while formerly studying the diseases of children under MM. Trousseau and Blache, he had been much struck by a fact which all observers must have sometimes remarked in young children, viz.: that a widely extended pneumonia is more readily cured than some examples of a much more limited character. Thus, while a case of double pneumonia yields to treatment in from nine to twelve days (the average period in early childhood,) another of far less extent, and not yet reaching hepatization, proves fatal in four or five days. On examining into these latter cases more closely, they were found to differ from the others in their mode of production, symptoms, and anatomical characters; and, on account of such difference, the author designates them as *mucous pneumonias.* They are found especially in vigorous stout children, whose mucous secretions are abundant, and usually commence in a cold or bronchial catarrh, and often during the first dentition. The child coughs for some days, with or without fever, but repeated auscultation only detects the signs of ordinary catarrh; when, at a certain point, the symptoms of pulmonary inflammation suddenly set in.

The pneumonia of infancy is recognised by the subcrepitant *râle* in lobular, and the *souffle* in lobar pneumonia, by fever, dyspnea, with movement of the alæ nasi and the development of a depression along the base of the chest, corresponding to the insertions of the diaphragm, and so characteristic as to be termed by Trousseau the *peripneumoniac furrow.* In *mucous pneumonia*, all these are present, but the inflammation is never lobar, and consequently no *souffle* is present; and a mucous *râle*, due to the abundant bronchial secretion, masks the subcrepitant *râle.* The symptoms are those of the suffocative catarrh of adults; but the autopsy exhibits also the alterations found in lobular pneumonia.

It is an affection of great danger, and one concerning which, owing to its seeming to be a mere bronchitis, a too favorable prognosis at first is almost constantly given. The pneumonia once becoming developed danger is very imminent. The free use of emetics forms the best medication, and, if given in the form of syrup, they are readily taken. For a child a year old the following formula may be employed—℞. Syrup. Ipecan., ℥i; Pulv. Ipecac., gr. ii.; Ant. Tart., ⅓ gr. A teaspoonful is given every ten minutes until five or six vomitings have occurred. The mixture is then suspended for several hours, and resumed in the same manner—a rather larger dose being now usually required in the intervals, some advantage may be gained by administering small doses of the white oxide of antimony and Kermes mineral in an emulsion, but this is merely adjuvatory. External revulsives may also be had recourse to, and Dr. Duclos entertains a high opinion, both in this and other forms of pneumonia in children, of the application of blisters to the legs, and encouraging suppuration afterwards by stimulating dressings. In spite of all treatment, however carefully directed, great numbers will succumb.—*Bulletin de Thérapeutique, tome* xxxvii, pp. 441–6.

